# Internalised stigma among people with mental illness in Africa, pooled effect estimates and subgroup analysis on each domain: systematic review and meta-analysis

**DOI:** 10.1186/s12888-023-04950-2

**Published:** 2023-06-29

**Authors:** Wondale Getinet Alemu, Clemence Due, Eimear Muir-Cochrane, Lillian Mwanri, Anna Ziersch

**Affiliations:** 1 College of Medicine and Public health, Flinders University, Adelaide, Australia; 2 Department of Psychiatry, College of Medicine and Health Sciences, University of Gondar, Gondar, Ethiopia; 3 School of Psychology, The University of Adelaide, Adelaide, Australia; 4 Torrens University Australia, Adelaide Campus, South Australia; 5 College of Nursing and Health Sciences, Flinders University, Adelaide, Australia

**Keywords:** Stigma, Perceived stigma, Internalised, Self-stigma, Mental illness, Africa

## Abstract

**Background:**

Internalisation of stigma occurs when people with a stigmatised attribute, such as a mental illness, supress negative but accepted societal attitudes. However, as far as is known, there is no comprehensive picture of the prevalence of and factors associated with, internalised stigma among people living with mental illness in Africa. This systematic review and meta-analysis provide new knowledge by examining the evidence on the prevalence of internalised stigma and associated factors among people living with mental illness in Africa.

**Methods:**

Using the population, intervention, comparison, outcome, and type of study (PICOT) approach, PubMed, Scopus, MEDLINE, PsycINFO, CINAHL, ScienceDirect, and Google Scholar were searched using a structured search comprising terms associated with mental health, mental illness, internalised stigma, and a list of all African countries. To evaluate paper quality, the Joanna Briggs Institute Quality Appraisal Checklist was used. Subgroup analysis with country and diagnosis was tested using a random-effect model, and bias was checked using a funnel plot and an inspection of Egger’s regression test. A p-value, OR and 95% CI was used to demonstrate an association.

**Results:**

The pooled prevalence of internalised stigma was 29.05% (25.42,32.68: I^2^ = 59.0%, p ≤ 0.001). In the subgroup analysis by country, Ethiopia had the highest prevalence of internalised stigma at 31.80(27.76,35.84: I^2^ = 25.6%, p ≤ 0.208), followed by Egypt at 31.26(13.15,49.36: I^2^ = 81.6%, p ≤ 0.02), and Nigeria at 24.31(17.94,30.67: I^2^ = 62.8%, p ≤ 0.02). Based on domains of internalised stigma, pooled prevalence was stigma resistance: 37.07%, alienation: 35.85%, experience of discrimination: 31.61%, social withdrawal: 30.81% and stereotype: 26.10%. Experiencing psychotic symptoms (1.42(0.45,2.38)), single marital status (2.78(1.49,4.06)), suicidal ideation (2.32(1.14,3.49)), drug nonadherence (1.5(-0.84,4.00)), poor social support (6.69(3.53,9.85)), being unemployed (2.68(1.71,3.65)), and being unable to read and write (3.56(2.26,4.85)) were identified as risk factors for internalised stigma.

**Conclusions:**

Internalised stigma is common among people suffering from mental illnesses in Africa. This review determined that 29% of the sample population had elevated internalised stigma scores, and there were variations by country. People experiencing mental illness who have a single marital status, suicidal behaviours, poor social support, unemployed and have poor literacy levels were at a higher risk of internalised stigma. The finding points to populations that require support to address internalised stigma and improve the mental health outcomes.

**Supplementary Information:**

The online version contains supplementary material available at 10.1186/s12888-023-04950-2.

## Introduction

Mental illness affects one in six adults [1], and accounts for 11% of the global burden of disease [2]. Mental illnesses are defined in this paper as serious psychiatric disorders characterized by “a change in cognition, emotion, or behaviour that are associated with distress/or poor performance” [3, 4]. Mental illnesses interfere with daily life, including education, work and relationships, and negatively affect the quality of life and wellbeing [5]. People with mental illnesses also face substantial challenges associated with these illnesses including negative societal beliefs regarding mental ill-health [6].

Stigma towards mental illness is a process of devaluation and unfavourable stereotypes of individuals who are diagnosed with mental illness [7], and refers to negative attitudes or behaviors towards an individual on the basis of their condition [8–11]. Globally, people with mental illness are often stigmatised because of other people’s (lack of) knowledge, attitudes, and behaviours towards them. Importantly, stigma can also become internalized, whereby people with mental illness internalize such negative social attitudes [12]. Stigma can have significant negative impacts on those living with mental illness, including poverty, victimization, and poorer quality of life [13, 14]. In terms of definitions of internalised stigma, the internalised stigma of mental illness scale (ISMI) is the most widely used measure in research on this topic [15]. Measures include several domains that help define the scope of internalised stigma, including alienation, stereotype endorsement, discriminatory experience, social withdrawal, and stigma resistance.

In general, studies show that internalised stigma is linked to lower quality of life across all World Health Organization quality of life dimensions (physical, psychological, environmental, and social) (WHOQOL-Brief) [16–19]. Furthermore, higher levels of internalised stigma are associated with more severe psychiatric symptoms, poor adherence to treatment, decreased mental health service utilization [20–25], and help seeking and recovery [26]. For example, worldwide, the World Health Organization has estimated that 75% of people with mental illness do not seek professional help [27] and internalised stigma is one of the most significant barriers to people with mental illnesses in receiving timely treatment [28].

The occurrence of internalised stigma in people with mental illnesses has been found to vary by geographic location and country. For example, the US study found the prevalence of internalised stigma to be 36% [29], in Ethiopia the prevalence has varied between 28% and 84% [30], and in Nepal it is 54% [31–33]. Country variations have also been found in the domains of internalised stigma. For example, a Poland study indicated that the alienation domain had a high score, while the stereotype endorsement had the lowest [34]. On the contrary, a study from Ethiopia found that alienation had the highest score [35], but a Japanese study indicated high stigma resistance and low stereotype endorsement [36]. In addition, stigma appears to vary among care settings. For example, 45% of participants receiving community-based care in India reported stigma, compared with 34% of those receiving hospital-based care [37].

Stigma also appears to vary with diagnosis. For example, in Ethiopia, 34% of participants with a depressive disorder reported experiencing internalised stigma [38], compared with 84–97% of those with schizophrenia [39, 40]. Other factors have also been identified as associated with internalised stigma including being single [12, 41], having an illness greater than or equal to 2 years of duration [12], history of suicidal attempt [12], non-adherence to treatment [12, 32], poor social support, poor quality of life [12, 42–45], lower levels of self-esteem [14, 43], lower levels of social support and the lack of formal education [43, 45, 46].

The differences in prevalence and associated factors of stigma observed in different countries may stem from differences in methodological approaches, sample sizes, sample characteristics, the type of stigma, attitudes to mental illness, and study regions/settings. This review focused on both systematic review and meta-analysis, pooled effect estimates of different associated factors based on diagnosis of mental disorder, country, and domains of stigma. The current review and meta-analysis answer the following questions:

1. How common is internalised stigma among those who experience mental illness in Africa?
2. What is the magnitude of internalised stigma by domains among people experience mental illness?
3. What factors contributed to internalised stigma among those experience mental illness?

## Methods

### Protocol registration and publication

This systematic review and meta-analysis were registered on the International Prospective Register of Systematic Reviews (PROSPERO) with the number **CRD 42,022,287,525**[47]. The protocol followed the Preferred Reporting Items for Systematic Reviews and Meta-Analysis (PRISMA) guidelines for methodological uniformity of the review process [48]. The Meta-analysis of Observational Studies in Epidemiology guidelines were also followed [49].

### Sources and data search strategy

To design the search strategy for this systematic review, the PICOT approach (*Eriksen and Frandsen, 2018*) was employed as follows [50]: The P (population of interest) was made up of people experiencing mental illness in Africa. There was no intervention (I) required for this review, no comparison (C) or control groups. Finally, the internalised stigma of mental illness scale was used to measure the outcome (O) (ISMI) and all empirical studies that published primary data pertinent to the study topics were considered (T) by the type of study. We used electronic and manual searches to identify articles for the systematic review and meta-analysis. PubMed, Scopus, MEDLINE, PsycINFO, CINAHL, ScienceDirect, and Google Scholar were searched to access data, with the databases chosen in partnership with a university research librarian. The key search terms were ((Stigma OR prejud* OR discriminat* OR alienat* OR stereotyp*) OR AB (Stigma OR prejud* OR discriminat* OR alienat* OR stereotyp*) AND ((Mental N3 (Health OR illness OR disorder*) OR AB ((Mental N3 (Health OR illness OR disorder*)). The search was conducted on 23/03/2022.

#### Inclusion criteria

Only correlational studies with the same study design were included in the final analysis that is cross-sectional, reporting the prevalence and associated factors of internalised stigma in Africa and articles were included without restriction by year of publication, because there simply were no other designs found in our search. Participants in the study had to be living in Africa and experiencing a mental illness – diagnosable conditions as per the DSM 5. Papers had to be published in English and in a peer-reviewed journal no time restriction was done on publication year.

#### Exclusion criteria

We excluded duplicates, reviews, commentaries, interventional studies, and studies not conducted in an African country. Grey literature was also excluded.

### Study screening and selection

Initially, research papers obtained from the specified databases were imported into EndNote X20 and then transferred to Covidence. Duplicates were removed using Covidence. Titles and abstracts were screened, followed by full texts. In the case where studies were found in databases but did not have full information, further details were sought from corresponding authors via email.

### Quality assessment and risk of bias

The methodological quality of the papers was assessed using the Joanna Briggs Institute Critical Appraisal checklist [51]. The quality assessment scores were converted to percentages to provide an overall score (0–10: poor,11–20: slight,21–40: fair,41–60: moderate,61–80: substantial,81–100: perfect). The JBI tool uses the following criteria: Was the sample frame appropriate to address the target population? Were study participants sampled in an appropriate way? Was the sample size adequate? Were the study subjects and the setting described in detail? Was the data analysis conducted with sufficient coverage of the identified sample? Were valid methods used for the identification of the condition? Was the condition measured in a standard, reliable way for all participants?, Was there appropriate statistical analysis?, Was the response rate adequate, and if not, was the low response rate managed appropriately?) which were then included in the study [52].

### Data extraction process

After eligible studies were identified, a Microsoft Excel spreadsheet with a prepared format was used for data extraction. Information was extracted as follows: author name/s, sample size and response rate, year of publication, region of study, and participant characteristics.

### Data analysis and publication bias

To explore prevalence of internalised stigma and stigma domains within the included studies, we calculated the logarithm of prevalence and standard error of logarithm of prevalence. Associated factors, variables’ odds ratios, the logarithm of odds ratio, and standard error of the logarithms of odds ratio were computed. Data were exported to Stata for analysis. The random analysis-effects model was used to show summary statistics, and heterogeneity among studies was examined using the I^2^ heterogeneity test and Q test [53]. The thresholds for I^2^ heterogeneity of 25%, 50%, and 75% used to indicate low, moderate ,severe heterogeneity respectively [54, 55]. The assumption of the random effects model, an estimate of random variation across studies was applied. A subgroup analysis and meta-analysis were performed considering the type of mental illness, diagnosis of disorder, study region, and country of the study. Small study bias was examined via an asymmetric funnel plot and objective inspection of Egger’s regression test [56]. Publication bias was declared if the funnel plot was asymmetrical or if Egger’s regression assumption test result was statistically significant (p < 0.05) [57, 58]. The pooled estimate prevalence and the pooled effects odds ratio were presented at a 95% confidence level. The results are described using narrative synthesis.

## Results

### Search outcomes

**Fig. 1:**
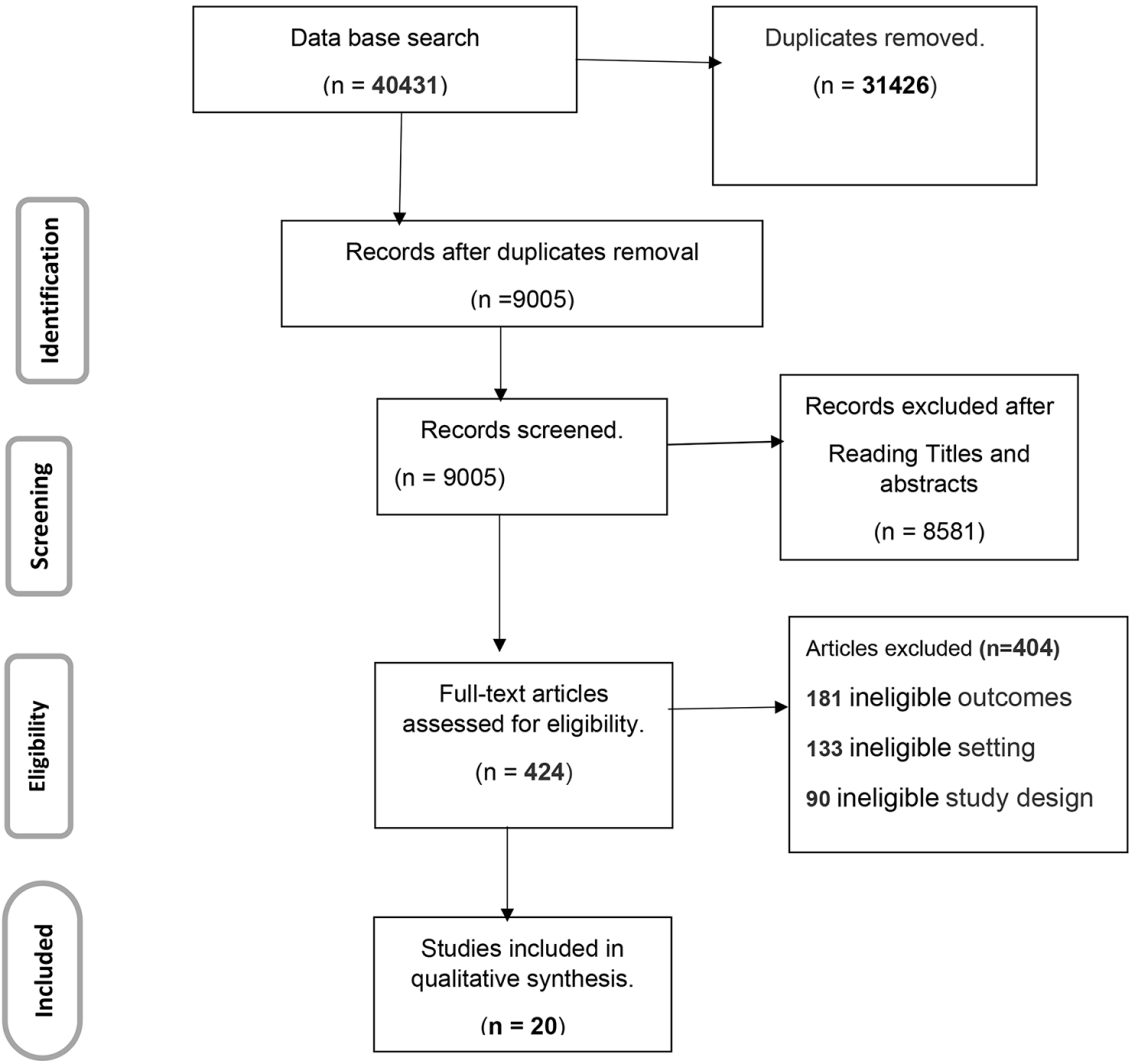
Prisma diagram that shows the selection of studies for the systematic review and meta-analysis of prevalence and associated factors of internalised stigma among patients with mental illness in Africa, 2022

A PRISMA flow diagram was used to present the selection processes and reasons for exclusion of papers (Fig. 1).

Meta-analysis of Observational Studies in Epidemiology guidelines [49] and the PRISMA reporting checklist were applied in reporting the study findings [48]. A total of 40,431 articles were found in the systematic literature search. of the available total, 31,426 articles were duplicates, and 8581 were assessed as irrelevant after screening the titles and abstracts, and they were excluded from the analysis. In addition, 404 articles were ineligible for reasons including: outcomes not being mental illness stigma, study setting out of Africa, or study design not meeting the eligibility criteria (for example interventional and qualitative studies). A total of 20 articles were assessed as eligible and were included for analysis. Included articles were cross-sectional studies conducted in Africa (Table 1).

**Table 1:** A descriptive summary of the 20 include studies

No	Author	Year	country	Study area	Sample size	R/rate (%)	The objective of the study	Domains of stigma	Tool	Stigma % (95% CI)	findings
1	Abah S [59]	2017	Nigeria	Nigeria	300	100	Internalized stigma and quality of life among outpatients’ schizophrenia in kaduna	Social withdrawal (16.7) Discrimination (14.1%) Alienation (13.9%) Stereotype (12.7%)	ISMI	27(16.8,37)	Unemployed (AOR = 2.39,95%CI,1.39,4.10) was associated with stigma
2	Abd El-SD [60]	2018	Egypt	Tanta	120	100	Relationship between insight, self-stigma, and level of hope among patients with Schizophrenia	Social withdrawal (30.8%) Discrimination (25.8%) Alienation (39.2%) Stereotype (40%) Stigma Resistance (20.8%)	ISMI	22.5(13,31.7)	
3	Abiodun O [44]	2010	Nigeria	Lagos	342	98.8	Correlates of self-stigma among outpatients with mental illness in Lagos, Nigeria		ISMI	21.6(12.5,30.7)	Unemployment (OR 3.85, 95% CI 2.55–7.00), poor social support (OR 10.82, 95% CI 4.85–24.84), longer duration of illness (OR 10.35, 95% CI 4.36–25.78) and having full insight into the illness (OR 4.23, 95% CI 2.16–8.76) was associated with internalised stigma.
4	Akinjola O [61]	2021	Nigeria	Lagos	320	100	Self-stigma in patients with schizophrenia in a psychiatry hospital in Lagos, Nigeria		ISMI	25.3(15.5,35)	
5	Alem E [62]	2017	Ethiopia	Dilla	317	100	Impact of self-stigma on quality of life of people with mental illness		ISMI	32.1(21,43)	QoL (OR = 0.041; 95% C. I: -0.065, -0.012).
6	Amany A [63]	2019	Egypt	Minia	100	100	Internalized stigma of mental illness and its relationship with self-esteem and social support among psychiatric Patients	Social withdrawal (60%) Discrimination (61%) Alienation (60%) Stereotype (65%) Stigma Resistance (7%)	ISMI	41(28.5,53.5)	
7	Babatunde F [45]	2018	Nigeria	Lagos	370	100	Internalized stigma in schizophrenia: cross-sectional study of prevalence and predictors	Social withdrawal (20.8%) Discrimination (24.1%) Alienation (22.4%) Stereotype (8.6%) Stigma Resistance (28.1%)	ISMI	16.5(8.5,24)	Lack of formal education (OR = 3.908), absence of good social support (OR = 0.387), high psychopathology based on the BPRS-18 (OR = 1.156) were predictors of stigma
8	Biksegn A [32]	2018	Ethiopia	Dilla	317	100	Internalized stigma among Patients with Mental Illness	Social withdrawal (37%) Discrimination (35.2%) Alienation (36%) Stereotype (30%) Stigma Resistance (40%)	ISMI	32.1(21,43)	Being female (AOR = 0.11, 95%CI 0.09, 0.65 0.02, Nonadherence (AOR = 0.45 0.67, 0.95 0.03)
9	Dereje A [41]	2012	Ethiopia	Addis Ababa	212	100	Internalized stigma among patients with schizophrenia in Ethiopia:		ISMI	46.7(33,60)	Rural residence (OR = 5.67; 95% CI = 2.30, 13.00; p < 0.001), single marital status (OR = 3.39; 95% CI = 1.40, 8.22; p = 0.019), psychotic symptoms (OR = 2.33; 95% CI = 1.17, 4.61; p = 0.016) were significant predictors of stigma
10	Eba A [64]	2020	Ethiopia	Jimma	300	98.7	Self‑stigma and medication adherence among patients with mental illness		ISMI	28(17.7,38)	0.091, p = 0.009) and living with kids and spouse (std. β = − 0.099, p = 0.038) were negatively associated with self-stigma. WHODAS score (β = 0.501, p < 0.001), number of relapses (std. β = 0.183, p < 0.01) and medication nonadherence (std. β = 0.084, p = 0.021).
11	Elias T [65]	2020	Ethiopia	St. Paul	235	90.4	Internalized stigma among patients with mood disorders in Ethiopia: a cross‑sectional facility‑based study	Social withdrawal (26.4%) Discrimination (27.7%) Alienation (54.5%) Stereotype (21.7%) Stigma Resistance (54.9%)	ISMI	31.5(20.5,42.5)	Females (std. β = 0.169 with P < 0.01), Adherence to medication (std. β = − 0.212, P < 0.01) history of suicidal attempt (std. β = 0.140), Being married (std. β = − 0.204), increment in age (std. β = − 0.200);
12	Endaylalu D [66]	2017	Ethiopia	Addis Ababa	114	100	Prevalence and associated factors of internalized stigma among patients with severe mental disorders:		ISMI	43(30,56)	Experienced discrimination, r = 0.743, p < 0.05. duration of time living with the illness, r = 0.367, p < 0.05 self-esteem, r-0.486, p < 0.05. general self-efficacy = − 0.671, p < 0.05.
13	Eshetu G [67]	2013	Ethiopia	Jimma	422	100	Self-stigma among people with mental illness:		ISMI	25.1(15,35)	Females (std. β = 0.11, P < 0.05) history of traditional treatment (std. β = 0.11, P < 0.05). level of education (std. β = −0.17, P < 0.01), supernatural causes of mental illness (std. β = 0.16, P < 0.01)
14	Liyew A [68]	2020	Ethiopia	Jimma	178	100	The lifetime prevalence and factors associated with relapse among mentally ill patients.		ISMI	30.3(19.5,41)	
15	Shegaye S [43]	2020	Ethiopia	Addis Ababa	418	98.8	The magnitude of internalized stigma and associated factors among people with bipolar disorder	Social withdrawal (26.1%) Discrimination (36.8%) Alienation (36.1%) Stereotype (17%)	ISMI	24.9(15,34.7)	unemployed (adjusted OR (AOR) = 2.3, 95% CI: 1.0 to 5.0), unable to read and write (AOR = 3.3, 95% CI: 1.05 to 10.7), poor social support (AOR = 5.3, 95% CI: 1.9 to 15.0), ≥ 4 previous hospitalisations due to bipolar disorder (AOR = 2.6,95% CI: 1.1 to 6.1) and low self-esteem (AOR = 2.4, 95% CI: 1.1 to 5.1) had a significant association with internalised stigma
16	Temilola J [16]	2014	Nigeria	Abeokuta	256	100	Self-stigma, quality of life and schizophrenia:	Social withdrawal (26.2%) Discrimination (25%) Alienation (24.6%) Stereotype (11.7%) Stigma Resistance (72.7%)	ISMI	18.8(10,27)	low educational level (χ*2* = 22.69, *p* < 0.001), unemployment (χ*2* = 15.9, *p* < 0.001), low income (χ*2* = 25.03, *p* < 0.001), source of income (χ*2* = 12.52, *p* = 0.007) and severity of psychopathology (*t* = 8.245, *p* < 0.001).
17	Victor M [69]	2016	Ghana	Kumasi	31	100	An explanatory model of psychosis: impact on the perception of self-stigma by patients in three sub-Saharan African cities		ISMI	20.7(11.9,29)	
18	Victor M [69]	2016	Kenya	Nairobi	30	100	An explanatory model of psychosis: impact on the perception of self-stigma by patients in three sub-Saharan African cities		ISMI	37.5(25.7,49)	
19	Victor M [69]	2016	Nigeria	Ibadan	24	100	Impact on the perception of self-stigma by patients in three sub-Saharan African cities		ISMI	42.1(29.7,54.6)	
20	Yadeta A [12]	2020	Ethiopia	Addis Ababa	415	98.1	Internalized stigma and associated factors among patients with the major depressive disorder at the Outpatient Department	Social withdrawal (35.2%) Discrimination (36.6%) Alienation (37.6%) Stereotype (30.7%)	ISMI	33.5()22,44.8	Being single (AOR = 2:54, 95% CI: 1.30, 4.95), having an illness greater than or equal to 2 years of duration (AOR = 3:21, 95% CI: 1.66, 6.19), history of suicidal attempt (AOR = 2:33, 95% CI: 1.35, 3.99), nonadherence to treatment (AOR = 2:93, 95% CI: 1.62, 5.29), poor social support (AOR = 4:72, 95% CI: 2.09, 10.64), and poor quality of life (AOR = 3:16, 95% CI: 1.82, 5.49) were significantly associated with high internalized stigma

ISMI- Internalized Stigma of Mental Illness Inventory tool to measure stigma

The quality of the articles was assessed by two reviewers (WGA&EMC) with the Joanna Briggs Institute checklist. Agreement between reviewers was reached from moderate to perfect agreement (80–100%), only, one article by *Victor M,2016 et al.* [69] has 8/9 level of agreement. Seventeen papers received quality assessment scores of 9/9 (Supplementary Table 1) and a further three scored 8/9.

### The pooled prevalence of internalised stigma

Twenty quantitative studies with a total of 6265 participants from five different African countries were included for final analysis. In terms of country of residence in Africa, 4365 individuals resided in Ethiopia ,1621 in Nigeria, 220 in Egypt, 31 in Ghana and 30 in Kenya. Across the studies, 2474 cases were diagnosed as general or unspecified mental illness, 2631 cases were diagnosed as schizophrenia, 82 cases were diagnosed as psychosis, 425 cases were diagnosed as depression, 418 cases were diagnosed as bipolar disorders, and 235 cases were diagnosed as other mood disorders.

**Fig. 2:**
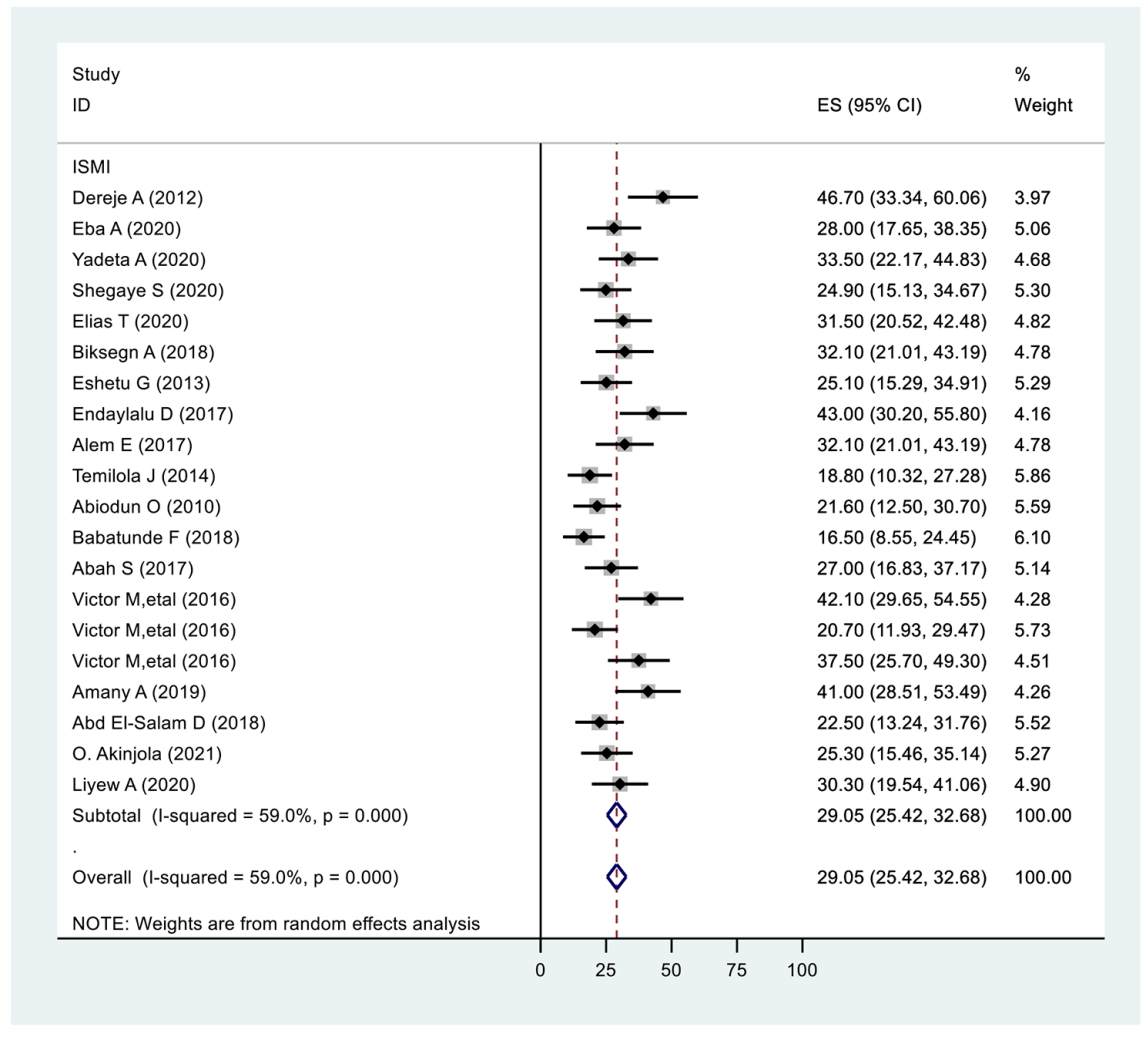
Forest plot of pooled prevalence of internalized stigma in Africa 2022 (*n* = 20)

All the included studies used the “Internalized Stigma of Mental Illness Inventory” to measure stigma, which derives scores ranging from 0 to 4, with a cut-off point 2.5. Across all studies, the mean internalised stigma score ranged from 2.51 to 4.00 coined as having internalised stigma and the pooled prevalence of internalised stigma was 29.05% (25.42,32.68: I^2^ = 59.0%, *p* ≤ 0.001) (Fig. 2).

### Subgroup analysis by diagnosis

**Fig. 3:**
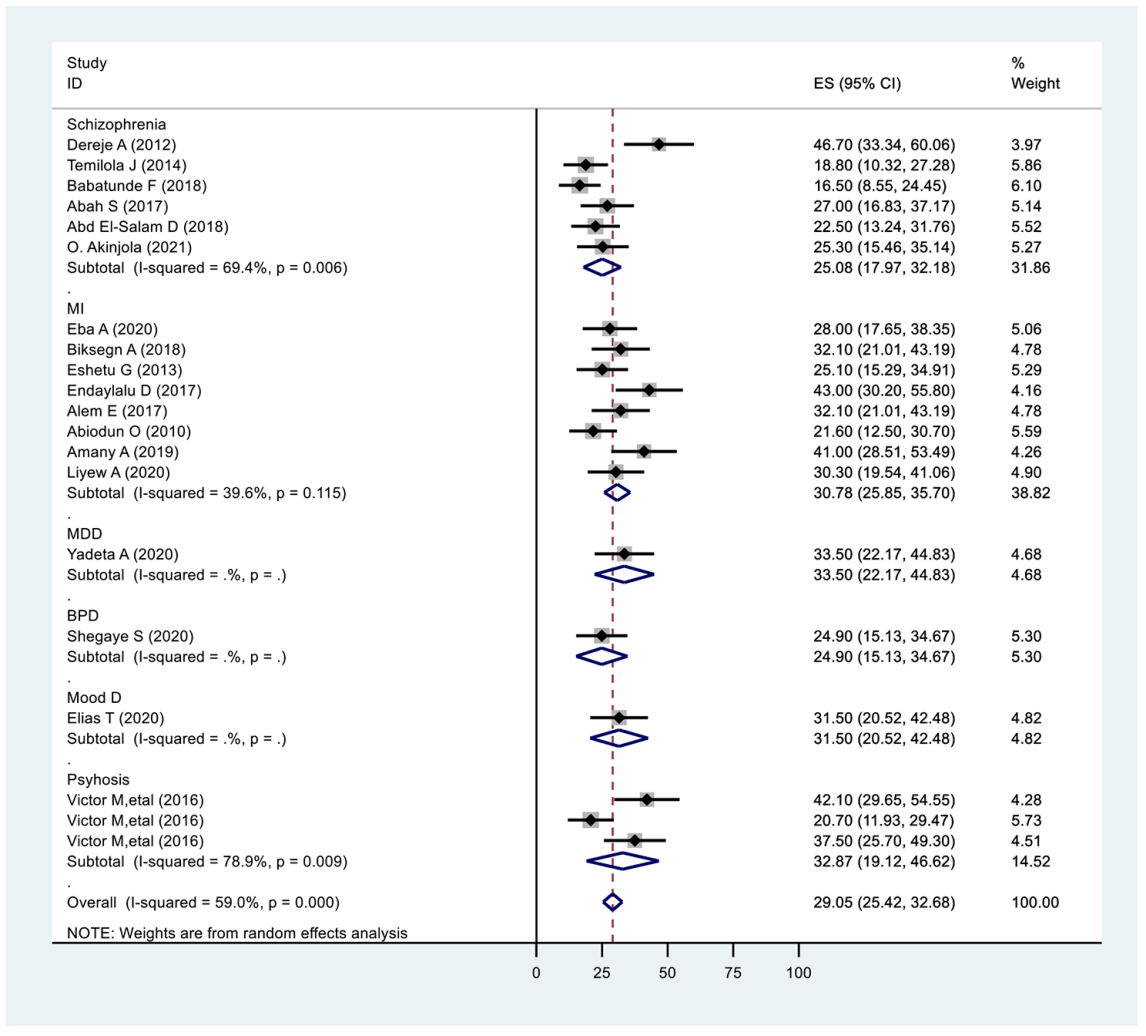
Subgroup analysis on prevalence of internalised stigma of mental illness based on diagnosis 2022 (n = 20)

In the sub-analysis random-effects model for specific mental illness, the pooled prevalence for diagnosis with schizophrenia was 25.08% (17.97,32.18: I^2^ = 69.4%, p ≤ 0.006), diagnosis with mental illnesses were 30.78% (25.85,35.70: I^2^ = 39.6%, P ≤ 0.115), and diagnosis with psychosis were 32.87% (19.12,46.62: I^2^ = 78.9%, p ≤ 0.009) and (Fig. 3).

### Subgroup analysis by country

**Fig. 4:**
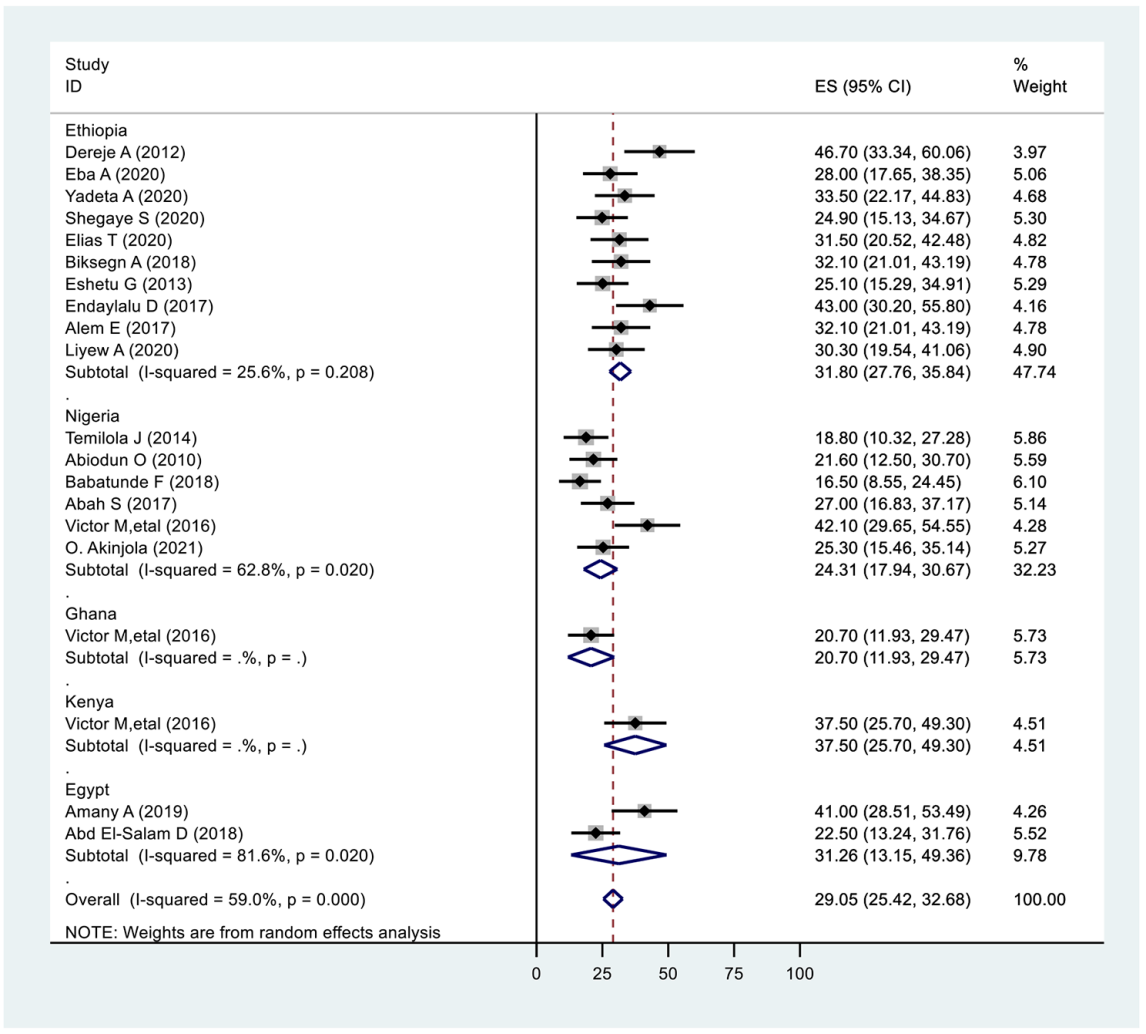
Subgroup analysis on the prevalence of internalized stigma of mental illness based on country 2022 (n = 20)

When analyzed by region, the highest prevalence of internalized stigma was found in Ethiopia, 31.80 (27.76,35.84: I^2^ = 25.6%, p ≤ 0.208); Egypt, 31.26(13.15,49.36: I^2^ = 81.6%, p ≤ 0.02); and Nigeria, 24.31(17.94,30.67: I^2^ = 62.8%, p ≤ 0.02). (Fig. 4).

### Publication bias

**Fig. 5:**
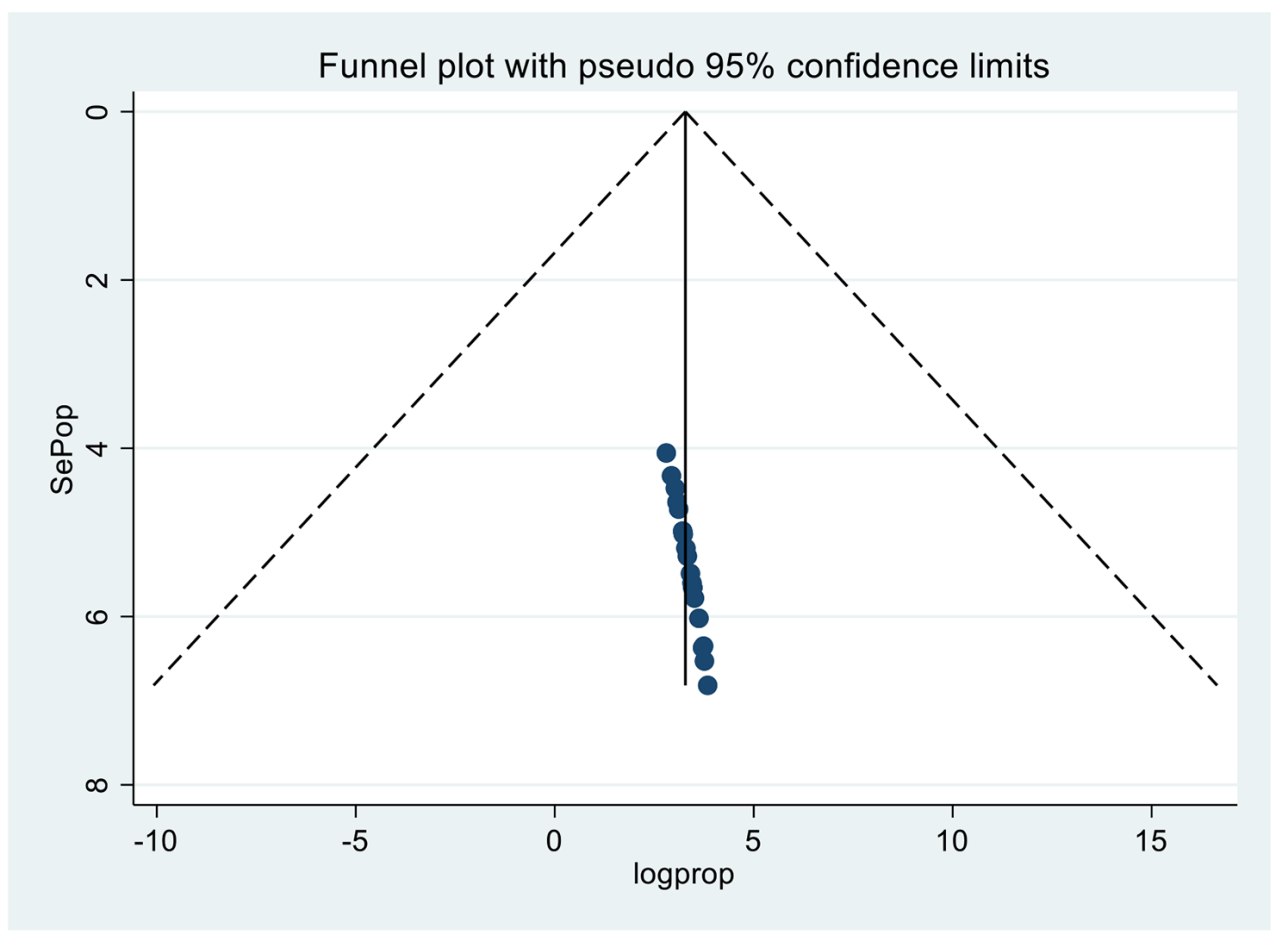
Funnel plot showing publication bias of prevalence of internalised stigma, a systematic review, and meta-analysis, in Africa,2022(n = 20)

There was no evidence of publication bias when the funnel plot was examined (Fig. 5). An Egger’s regression test confirmed heterogeneity, chi-squared = 46.38 (d.f = 19), I^2^ = 59.0%, Tau-squared(T2) = 39.78, Test of ES = 0: z = 15.7.

#### Results based on domain of stigma

**Fig. 6:**
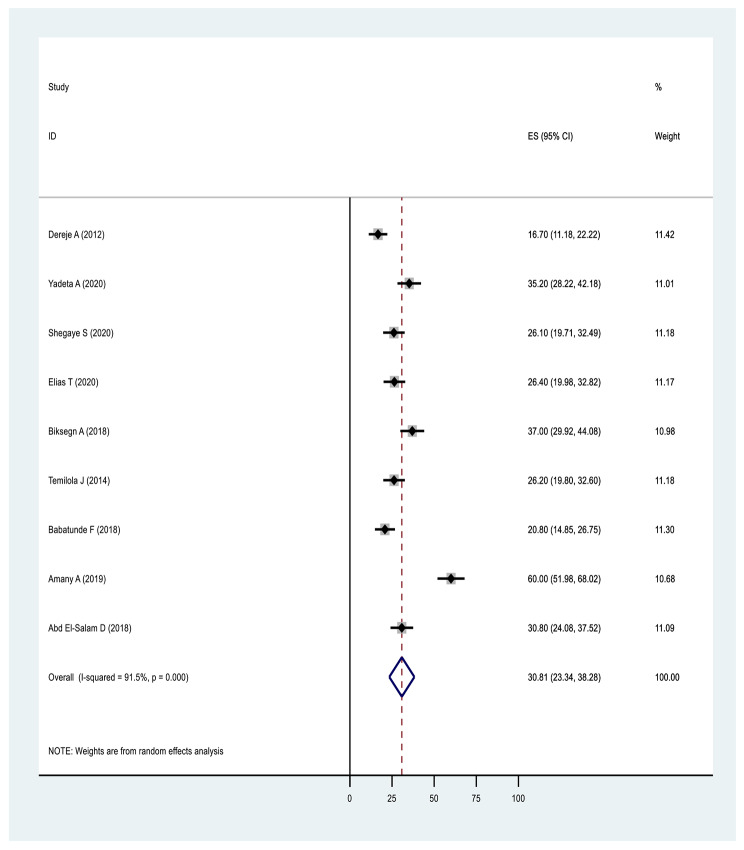
Forest plot of pooled prevalence of social withdrawal of internalised stigma in Africa 2022 (*n* = 9)

**Fig. 7:**
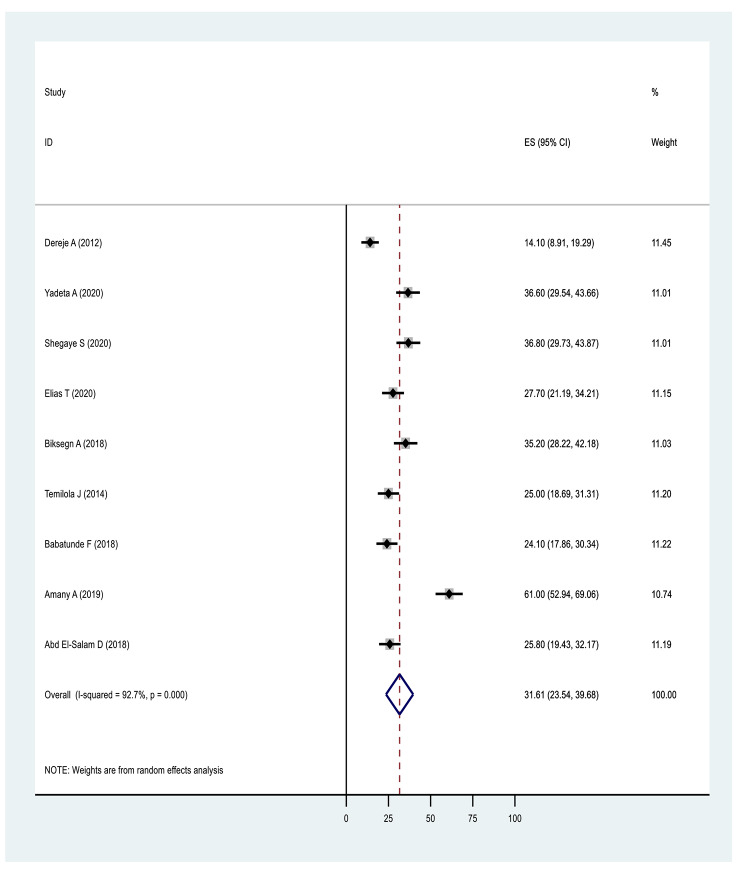
Forest plot of pooled prevalence of experience of discrimination of internalised stigma in Africa 2022 (n = 9)

**Fig. 8:**
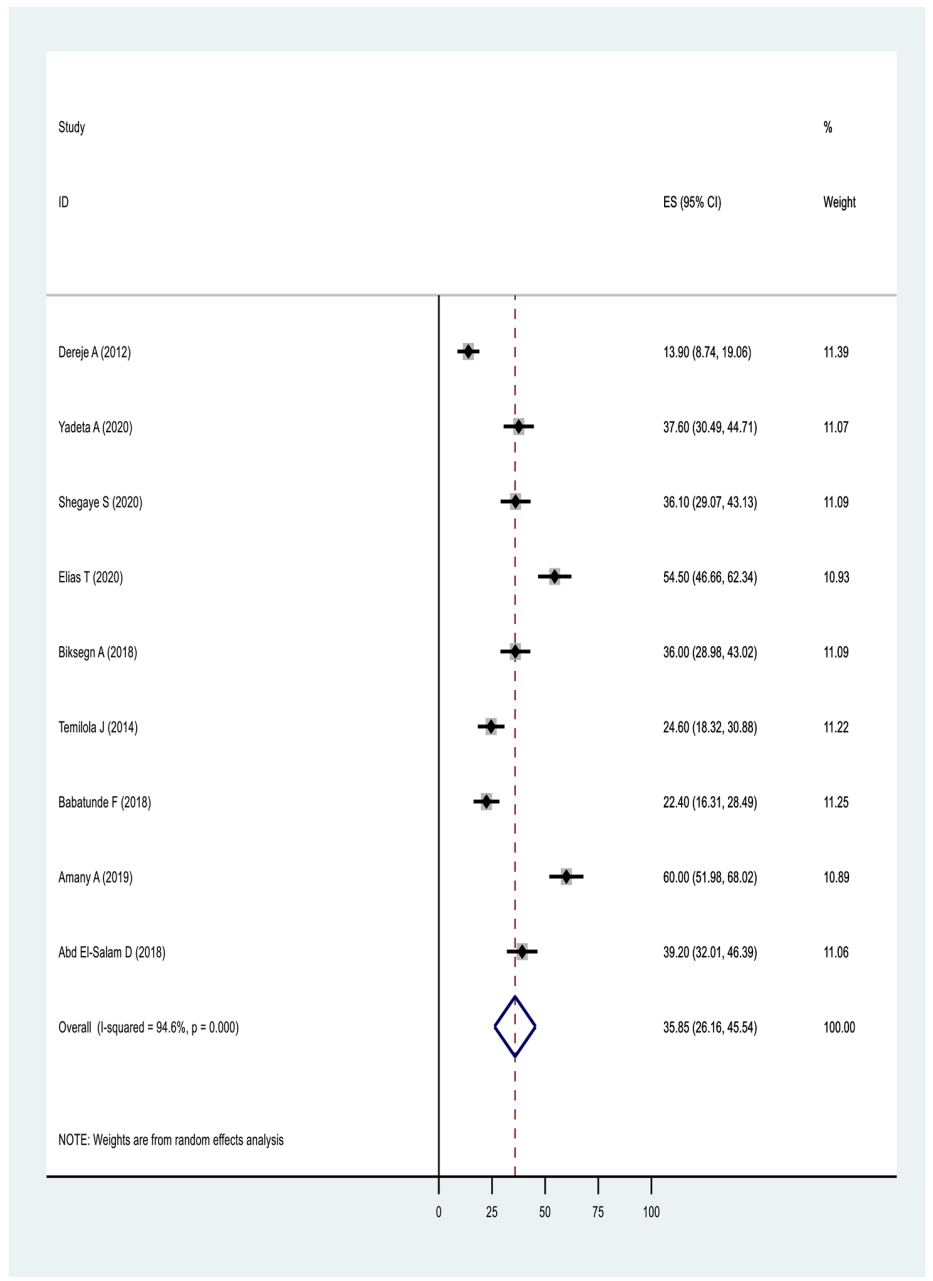
Forest plot of pooled prevalence of alienation of internalised stigma in Africa 2022 (n = 9)

**Fig. 9:**
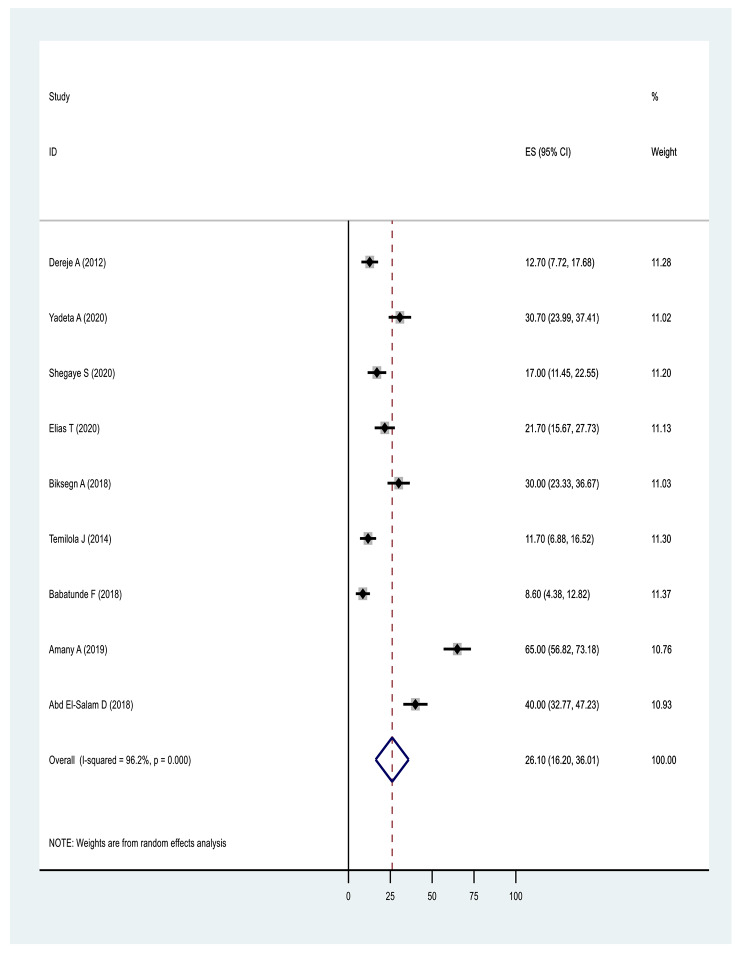
Forest plot of pooled prevalence of stereotype endorsement, of internalised stigma in Africa 2022 (n = 9)

**Fig. 10:**
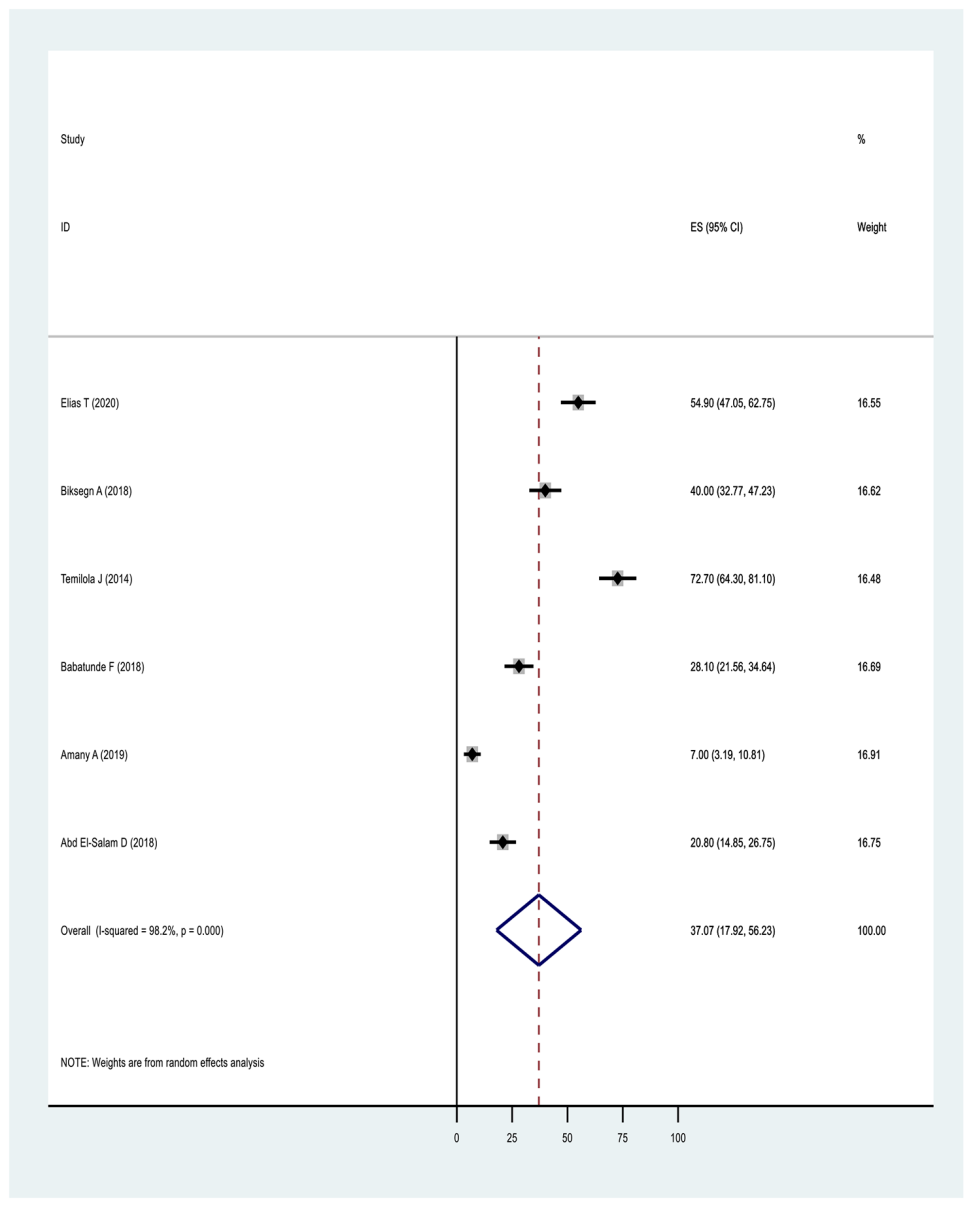
Forest plot of pooled prevalence of stigma resistance of internalised stigma in Africa 2022 (n = 6)

In terms of pooled prevalence for the domains of stigma, the six studies that measured stigma resistance received the highest score of 37.07(17.92,56.23: I^2^ = 98.2%, p ≤ 0001), followed by nine studies examining alienation with a score of 35.85(26.16,45.54: I^2^ = 94.6%, p ≤ 0001). Nine studies examining experience of discrimination scored 31.61(23.54,39.68: I^2^ = 92.7%, p ≤ 0.0001), nine studies measuring social withdrawal had a score of 30.81(23.34,38.28: I^2^ = 91.5%, p ≤ 0.0001) and nine studies examining stereotype had a score of 26.10(16.20,36.01: I^2^ = 96.20%, p ≤ 0.0001) (Figs. 6, 7, 8, 9 and Fig. 10).

#### Pooled effect estimates of Factors

We examined the socio demographic and other factors that predicted internalised stigma. The individual papers found the following: female gender [32], rural residence [41], single relationship status [12, 41], unemployed [43, 59, 61], unable to read and write [43, 45], low income [59], psychotic symptoms [41, 45], suicidal behaviour [12, 41], greater than two years since diagnosis [12], drug nonadherent [12, 32], presence of drug side effect/s [32]; previous hospital admission [43], longer duration of follow up [44], low self-esteem [43], no family support [32], poor social support [12, 43–45], poor quality of life [12], and full insight into the condition [44] were significantly associated with internalised stigma.

**Fig. 11:**
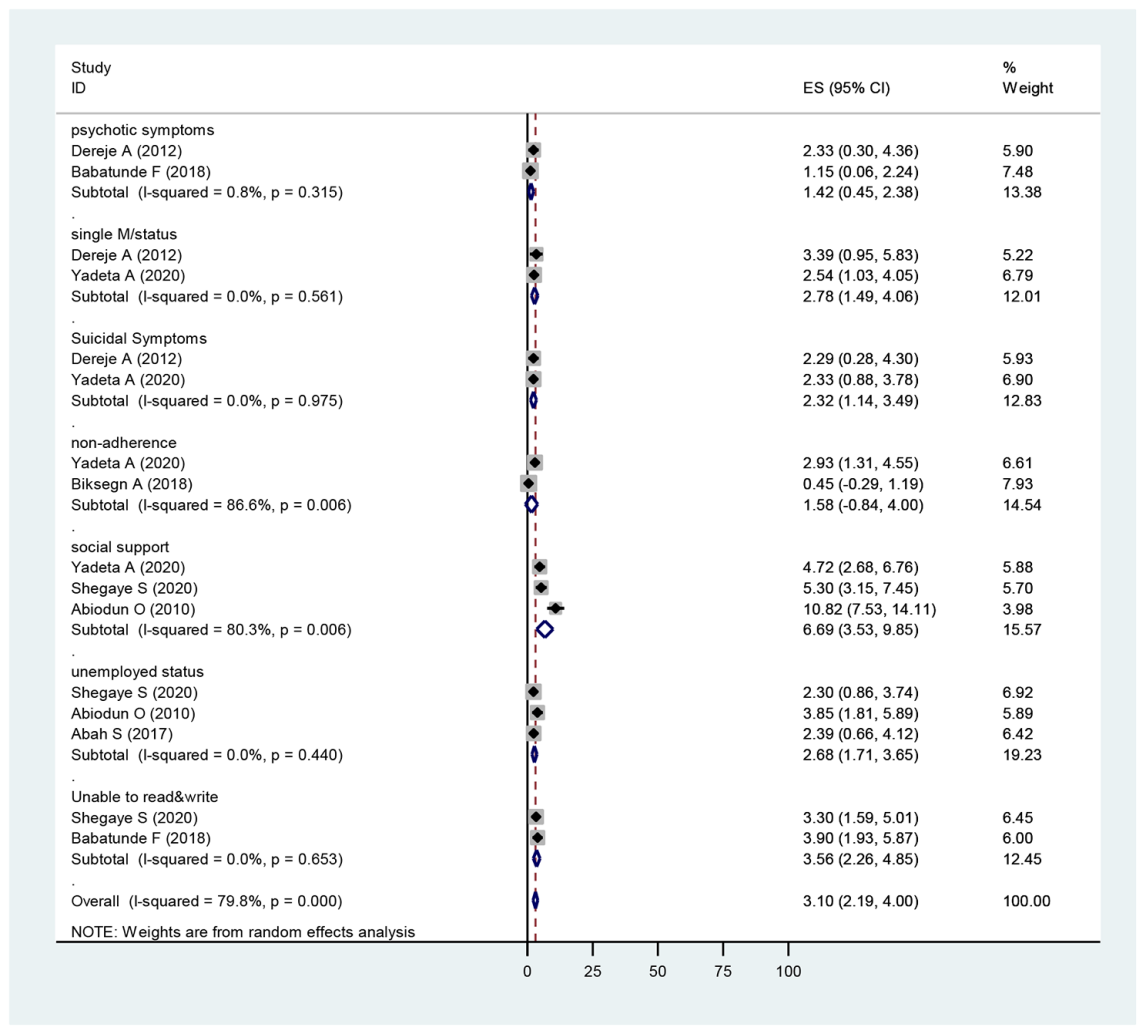
Forest plot of pooled effect estimate of different factors

The pooled analysis for these factors and where there were two or more papers, the following outcomes were found. Those who were single were 2.78(1.49,4.06), times more likely to report internalised stigma than those who were married [12, 41]. People who unemployed were 2.68(1.71,3.65) more likely to report internalised stigma than employed participants [43, 44]. There was a significant difference between educational status on developing internalised stigma, and those who were not able to read and write were 3.56(2.26,4.85) more likely to report internalised stigma than those who were able to read and write [43, 45]. See on (Fig. 11).

Pooled effect estimates examining psychotic symptoms found that there were no significant differences between patients with psychotic symptoms 1.42(0.45,2.38), than patients without psychotic symptoms [12, 41]. Participants with a history of suicidal ideation were 2.32(1.14,3.49) times more likely to report internalised stigma when compared to participants who did not have suicidal symptoms [12, 41].

There was no significant relationship between patients with drug nonadherence and internalised stigma 1.5(-0.84,4.00) as demonstrated by the pooled effect estimate [12, 32].

There was an increase in stigma associated with poor social support in the pooled analysis. Participants with poor social support were 6.69(3.53,9.85) times more likely the report internalised stigma than people who did have good social support [12, 43–45].

## Discussion

The major objective of this systematic review and meta-analysis was to determine the prevalence and pooled effect estimates of internalised stigma among people with a mental illness in Africa. Stigma and discrimination continue to be barriers to people in need of mental health services, obtaining help and fully recovery [23, 26]. Furthermore, there is limited synthesis of research examining internalised stigma amongst people with a mental illness in low and middle-income countries. In order to fill this gap, this systematic review and meta-analysis assessed the pooled prevalence, pooled effect estimates and subgroup analysis on domains of internalised stigma among African people living with a mental illness. Results presented were based on study area (country), type of mental illness, and associated factors.

In the current study, the overall pooled prevalence of internalised stigma was found to be 29%. This is consistent with a previous meta-analysis which found 31.3% stigma in patients with a severe mental illness [70]. However, these figures were higher than an analysis of 15 different countries which found a stigma prevalence of 22.2% in patients with severe mental illnesses [71]. However, as stigma is pervasive across the world, a previous review found the prevalence of stigma among patients with mental illnesses to be 39.7% in South East Asia and 39% in the Middle East [70]. Subgroup analysis based on country of study in the current review found that Ethiopia had the highest pooled prevalence of at 31.80% followed by Egypt which had a prevalence of 31.26% and Nigeria with the prevalence of 24.31%. Studies about stigma of mental illnesses in African countries have been carried out in a limited number of areas. These studies have reported varying and high internalised stigma prevalence including Kenya 38% [69], Ghana 21% [69], Ethiopia 47% [41], Nigeria 22% [44], and Egypt 23% [60] respectively. These differences in the magnitude of internalised stigma may be related to the level of awareness of mental illnesses among individuals with the problem, as well as variations with the mental health workforce and literacy related issues. For instance, the number of mental health professionals in Ethiopia is five times less than the number of health care professionals globally, indicative of fewer health care professionals among low-income countries [72, 73], for which Ethiopia is not exceptional. This may possibly mean that in these countries, patients may not receive adequate level of education to increase the awareness about their illness and may lead to the development of self-stigmatisation.

One of the most significant findings from this meta-analysis was the subgroup analysis based on domains of internalised stigma, this analysis provided scores as follows: stigma resistance 37% (from six studies), alienation 36% (from nine studies), experience of discrimination 32% (from nine studies), social withdrawal 31% and stereotype 26% (both from nine studies). These results agree with the findings of other systematic review domains conducted in Europe, North America, Australia, and Asia, in which scores were reported as follows: Alienation shame 49%, Stereotype endorsement 27%, discrimination 35%, Stigma resistance 52% [74]. However, as this reporting was based on a single multi country study (Europe, North America, Australia, and Asia) and. There was no reporting of each domain of internalised stigma per country, more research on this topic needs to be undertaken to show the magnitude of each domain and its contribution on stigma in different countries.

In terms of factors associated with internalised sigma, none of previous studies have demonstrated that the sociodemographic variables had consistent significant relationship with internalised stigma [71]. In contrast, the findings of this review found several sociodemographic factors were significantly associated with internalised stigma. For example, having a single marital status was 2.78 times more likely to be stigmatized than being married. We also found that those who were unemployed were 2.68 times more likely to report developing internalised stigma than employed participants. The lack of resources, poverty, and other economic obstacles associated with a lack of employment have been cited as stigmatising factors and obstacles to mental health care in African countries [75]. These factors have also been reported to contribute to internalised stigma regarding mental illness [76]. We conclude that the differences between our study and the one by Dunn and colleagues above [71], may relate to the differences in the number of articles included in the review and our focus of study which was studies conducted only in African countries.

The current study also found that there was a significant difference between those who were educated and those who were not. Patients who developed internalised stigma were not able to read and write, and their likelihood of developing internalised stigma was 3.56 time more than someone who was able to read and write. Education has been linked to stigma on other conditions such as infectious diseases [77, 78]. Education may affect stigma through a lack of awareness and incorrect information about mental illness, contributing to internalised stigma. The relationship between education and stigma has been investigated, but causal correlations are multifactorial and notoriously difficult to demonstrate, yet higher levels of education have been linked to good mental health which may indicate that poor knowledge negatively contribute to mental health stigma [79]. Further studies have shown that one of the most obvious predictors of good outcomes in life, including employment, wealth, and social status is education, and as a result, education has a high degree of predictive value for better health and wellbeing outcomes [80].

In terms of conditions and condition management, previous systematic reviews have found that schizophrenia was associated with a high level of self-stigma, including 56% from studies of Europe, North America, Australia, and Asia [74]. However, in our finding the subgroup analysis showed that the results of pooled prevalence of stigma was not different in each mental illness, but participants with a diagnosis of schizophrenia had a lower prevalence of 25% compared to other diagnoses such as psychosis that had an internalised stigma of 32% and Mental illness as diagnosis internalised stigma of 30%. This stands in contrast to data that indicates stigma is most severe for those with a diagnosis of schizophrenia. In this systematic review and meta-analysis stigma of schizophrenia was 25.08% (17.97,32.18) which is low when compared to previous research. In previous reviews there has been a strong relationship between stigma and having psychotic symptoms [71]. However, in this meta-analysis there was no significant association between internalise stigma and presence of psychotic symptoms on people with mental illness.

To date, little evidence has been found associating suicidal symptoms and internalised stigma. In this meta-analysis, studies indicated a significant positive correlation between suicidal ideation and internalised stigma, where people with a history of suicidal ideation were 2.32 times more likely to develop internalised stigma than those who did not [12, 41]. This is consistent with research around other stigmatised conditions such as HIV-related stigma, and links with suicidal ideation [81, 82].

In the current review there was no difference between patients with drug adherence and non-adherence. These results a contradict other studies which have demonstrated a negative correlation between drug non-adherence and internalised stigma [70, 71]. Additionally, a study using the meta-analysis data around the world, revealed a weak association between HIV related stigma and poor treatment adherence of [83].

In terms of social support, this meta-analysis findings mirror findings from previous studies that have examined the effect of social support on internalised stigma. In the pooled estimate there was an increase in stigma associated with poor social support, where those with poor social support were 6.69 times more likely of developing internalised stigma than people who did have good social support. This result is consistent with the research showing a relationship between HIV stigma, another stigmatising condition, and poor social support [81, 83]. Social support may provide greater emotional assistance, decrease symptoms associated with mental illness [84] and improve emotional wellbeing, which is crucial for reducing mental distress including stigma [85–88]. In contrast, poor social support from the government or family and friends could be perceived by the person as discrimination associated with their illness [88] and may lead to developing internalised stigma. Qualitative research has likewise indicated that those with mental illness who do not have good social support experience a solitary lifestyle, loneliness and feel stigma related to mental health more severely [89]. Isolation and mental health have been associated with internalised stigma and vice versa [90].

Overall, our findings show high rates of internalised stigma, and variations across countries, conditions, including sociodemographic and other factors. It has been alluded that variations in cultural views on stigma within countries, levels of poverty and access to mental health services, may be contributory factors to these differences [91]. In some countries including Ethiopia, a mental illness may not be regarded as a life-threatening condition [91, 92]. As such, policymakers and planners and health care providers may not prioritised mental health care and treatment [93, 94]. The lack of awareness and priority setting can be a significant determinant of poor mental health outcomes for populations affected by these conditions. As such improving health care access by government is an important area for future, including outreach programs and mental health awareness-raising activities that could help lessen stigma and enhance social outcomes for those with severe mental illness [95] and psychoeducational interventions, cognitive-behavioural interventions, mainly aimed at modifying self-stigmatising beliefs; interventions focused on the revelation of mental illness [96]. Moreover, building the capacity of policy makers, health care providers and strengthening the mental healthcare system and governance should be a priority in African countries such as Ethiopia and Egypt where internalised stigma was found to be significantly high.

### Strengths and limitations of the study

This systematic review and meta-analysis showed pooled prevalence in domains of stigma and examined a range of factors associated with internalised stigma. However, there are certain limitations considerations to be aware of. First, there are limitations in the literature itself where some factors, such as quality of life of participants, participants’ sex, residency, duration of diagnosis, previous hospital admission, patients’ self-esteem, family support, the presence of drug side effects, longer duration of follow-up, insight, and participants income, were not reported in adequate number to run meta-analysis to see whether they had effect on stigma and considered as predictors of internalised stigma. Future research should consider the effects of these influencing factors on the prevalence of internalised stigma, which could then be synthesised. Second, it is possible that some research reporting the prevalence of stigma were overlooked by our search approach as only English-language articles were included for analysis. Third, some diagnoses including mood disorder, depression, and bipolar disorder were represented by a single study. As such, it was difficult to ascertain the difference from another diagnosis. Fourth, we have noted the issue of study heterogeneity in the study limitations, factors found to be influencing stigma have been explored in several studies but for the pooled estimates for conditions with a small number of studies for pooled effect estimates are female gender, rural residence, greater than two years since diagnosis, having low income, presence of drug side effect/s, previous hospital admission, longer duration of follow up, low self-esteem, no family support, poor quality of life, and having full insight into their condition are represented with single studies. In addition, most of the included papers were from specific African countries, and only single studies in some from countries despite there being no restrictions on the inclusion or language. Given the small number of papers we decided to go ahead with the analysis despite the heterogeneity levels, but that some of the results need to be interpreted with caution. Furthermore, because all the studies included in this review were cross-sectional, the outcome variable may have been influenced by confounding variables. The current findings are based on bivariate cross-sectional data, and as such there are significant limitations in drawing conclusions about the direction and causality of the association between stigma and associated factors from being drawn.

## Conclusion

This systematic review and meta-analysis revealed that almost one third of patients with mental illness experience internalized stigma. Therefore, we can conclude that internalized stigma is common among people suffering from mental illness in Africa. The pooled prevalence rate also varies among domains of stigma, stigma resistance, alienation, discrimination experience, social withdrawal, and stereotype are ranked from highest to lowest in terms of internalised stigma. This indicates that a person centred approach should be advised for people living with a mental illness. Several risk factors relating to patients and mental illness are contributing for stigma. As such, emphasis should be given for patients with single marital status, having suicidal behaviour, poor social support, unemployed status, and unable to read and write as factors associated with a pooled effect estimate of internalised stigma. We recommend more representative samples be used in future research that concentrates on a more precise diagnosis. Future meta-analysis that focusses on both quantitative and qualitative studies address stigma and mental illness are also desperately needed.

## Electronic supplementary material

Below is the link to the electronic supplementary material.

Supplementary Material 1

Supplementary Material 2

Supplementary Material 3

## Data Availability

All data generated or analysed during this review are included in this published article and its supplementary information files.

## Abbreviations

AOR: Adjusted odds ratio
COR: Crude odds ratio
CI: Confidence interval
MOOSE: Meta-analysis of observational studies OR:Odds
SPSS: Statistical Package for Social Science
WHO: World Health Organization.
