# Supplementary material for: Internalised stigma among people with mental illness in Africa, pooled effect estimates and subgroup analysis on each domain: systematic review and meta-analysis
Source: BMC Psychiatry. 2023 Jun 29;23:480. doi: 10.1186/s12888-023-04950-2 (PMC10308748; doi:10.1186/s12888-023-04950-2)
Supplement: Supplementary file 2 — Supplementary Material 2 [file 12888_2023_4950_MOESM2_ESM.docx]

**Appendix I:**

**Search terms**

((Schizo* OR anxiety OR depression OR depressive OR bipolar OR paranoia OR psychosis OR "affective disorder" OR “Stress disorders” OR “trauma and stressor related disorders” OR “psychological trauma” OR “post-traumatic stress” OR “post-traumatic stress disorder” OR PTSD OR “traumatic disorders” OR “personality disorders” OR “somatization disorders”)) OR AB ((Schizo* OR anxiety OR depression OR depressive OR bipolar OR paranoia OR psychosis OR "affective disorder" OR “stress disorders” OR “trauma and stressor related disorders” OR “psychological trauma” OR “post-traumatic stress” OR “post-traumatic stress disorder” OR PTSD OR “traumatic disorders” OR “personality disorders” OR “somatization disorders”)) AND((Africa OR Algeria* OR Angola* OR Benin OR Botswana* OR "Burkina Faso " OR Burundi* OR " Cabo Verd* " OR " Cape Verd* " OR Comoros*OR Cameroon* OR " Central African” OR Chad* OR Congo OR " Cote d'Ivoire " OR " Ivory Coast " OR Djibouti* OR Egypt* OR Eritrea*OR “Equatorial Guinea” OR” Eswatini* OR Ethiopia* OR Gabon* OR Gambia* OR Ghana* OR Guinea* OR “Guinea Bissau” OR Kenya* OR Liberia* OR Libya* OR Lesotho* OR Madagascar* OR Malawi* OR Mali* OR Mauritania* OR Mauritius* OR Morocco* OR Mozambique OR Namibia* OR Niger* OR Nigeria* OR Rwanda* OR “Sao Tome” OR Senegal* OR Seychelles* OR " Sierra Leone " OR Somalia* OR “South Africa” OR “South Sudan” OR Sudan* OR Tanzania* OR Togo* OR Tunisia* OR Uganda* OR Zambia* OR Zimbabwe*)).
