# Supplementary material for: Internalised stigma among people with mental illness in Africa, pooled effect estimates and subgroup analysis on each domain: systematic review and meta-analysis
Source: BMC Psychiatry. 2023 Jun 29;23:480. doi: 10.1186/s12888-023-04950-2 (PMC10308748; doi:10.1186/s12888-023-04950-2)
Supplement: Supplementary file 3 — Supplementary Material 3 [file 12888_2023_4950_MOESM3_ESM.docx]

| Study | Agreement% | Kappa value | Level of agreement | JBI Checklist (0 to 9 scale) |
| --- | --- | --- | --- | --- |
| 1. Dereje A,2012 | 100 | 1 | perfect | 9 |
| 1. Eba A,2020 | 100 | 1 | perfect | 9 |
| 1. Yadeta A,2020 | 100 | 1 | perfect | 9 |
| 1. Shegaye S,2021 | 100 | 1 | perfect | 9 |
| 1. Elias T,2020 | 100 | 1 | perfect | 9 |
| 1. Biksegn A,2018 | 100 | 1 | perfect | 9 |
| 1. Eshetu G,2013 | 100 | 1 | perfect | 9 |
| 1. Endaylalu D,2017 | 100 | 1 | perfect | 9 |
| 1. Alem E,2017 | 100 | 1 | perfect | 9 |
| 1. Temilola J,2014 | 100 | 1 | perfect | 9 |
| 1. Abiodun O,2010 | 100 | 1 | perfect | 9 |
| 1. Babatunde F,2018 | 100 | 1 | perfect | 9 |
| 1. Abah S,2017 | 100 | 1 | perfect | 9 |
| 1. Victor M,2016 | 80 | 0.81 | substantial | 8 |
| 1. Victor M,2016 | 80 | 0.81 | substantial | 8 |
| 1. Victor M,2016 | 80 | 0.81 | substantial | 8 |
| 1. Abd El S,2021 | 100 | 1 | perfect | 9 |
| 1. Amany A,2019 | 100 | 1 | perfect | 9 |
| 1. Akinjola O,2021 | 100 | 1 | perfect | 9 |
| 1. Liyew A,2020 | 100 | 1 | perfect | 9 |

Supplementary table 1. JBI Quality assessment scores of included papers.

## **DATABASE: Medline**

| 1. 1 | 1. prejudice/ or bias, implicit/ or social isolation/ or ostracism/ or social stigma/ or stereotyping/ |  |
| --- | --- | --- |
| 1. 2 | 1. (Stigma or prejud* or discriminat* or alienat* or stereotyp*).tw,kf. |  |
| 1. 3 | 1. or/1-2 |  |
| 1. 4 | 1. exp Mental Disorders/ |  |
| 1. 5 | 1. Mental Health/ |  |
| 1. 6 | 1. (Mental adj3 (Health or illness or disorder*)).tw,kf. |  |
| 1. 7 | 1. (Schizo* or anxiety or depression or depressive or bipolar or paranoia or psychosis or trauma or PTSD or mania).tw,kf. |  |
| 1. 8 | 1. ((affective or Stress* or personality or somatization or somatoform or mood) adj4 disorder).tw,kf. |  |
| 1. 9 | 1. or/4-8 |  |
| 1. 10 | 1. exp Africa/ |  |
| 1. 11 | (Africa* or Algeria* or Angola* or Benin or Botswana* or "Burkina Faso" or Burundi* or "Cabo Verd*" or "Cape Verd*" or Comoros* or Cameroon* or "Central African" or Chad* or Congo or "Cote d'Ivoire" or "Ivory Coast" or Djibouti* or Egypt* or Eritrea* or "Equatorial Guinea" or Eswatini* or Ethiopia* or Gabon* or Gambia* or Ghana* or Guinea* or Kenya* or Liberia* or Libya* or Lesotho* or Madagascar* or Malawi* or Mali* or Mauritania* or Mauritius* or Morocco* or Mozambique or Namibia* or Niger* or Nigeria* or Rwanda* or "Sao Tome" or Senegal* or Seychelles* or "Sierra Leone" or Somalia* or "South Africa" or "South Sudan" or Sudan* or Tanzania* or Togo* or Tunisia* or Uganda* or Zambia* or Zimbabwe*).tw,kf. |  |
| 1. 12 | 1. or/10-11 |  |
| 1. 13 | 1. 3 and 9 and 12 |  |

##

## **DATABASE: PsycINFO**

| Ovid MEDLINE(R) and Epub Ahead of Print, In-Process, In-Data-Review & Other Non-Indexed Citations, Daily and Versions <1946 to March 22, 2022> | | |
| --- | --- | --- |
|  |  |  |
| 1 | prejudice/ or bias, implicit/ or social marginalization/ or social stigma/ or stereotyping/ |  |
| 2 | (Stigma or prejud* or discriminat* or alienat* or stereotyp*).id. |  |
| 3 | or/1-2 |  |
| 4 | exp Mental Disorders/ |  |
| 5 | Mental Health/ |  |
| 6 | (Mental adj3 (Health or illness or disorder*)).tw,id. |  |
| 7 | (Schizo* or anxiety or depression or depressive or bipolar or paranoia or psychosis or trauma or PTSD or mania).tw,id. |  |
| 8 | ((affective or Stress* or personality or somatization or somatoform or mood) adj4 disorder).tw,id. |  |
| 9 | or/4-8 |  |
| 10 | exp Africa/ |  |
| 11 | (Africa* or Algeria* or Angola* or Benin or Botswana* or "Burkina Faso" or Burundi* or "Cabo Verd*" or "Cape Verd*" or Comoros* or Cameroon* or "Central African" or Chad* or Congo or "Cote d'Ivoire" or "Ivory Coast" or Djibouti* or Egypt* or Eritrea* or "Equatorial Guinea" or Eswatini* or Ethiopia* or Gabon* or Gambia* or Ghana* or Guinea* or Kenya* or Liberia* or Libya* or Lesotho* or Madagascar* or Malawi* or Mali* or Mauritania* or Mauritius* or Morocco* or Mozambique or Namibia* or Niger* or Nigeria* or Rwanda* or "Sao Tome" or Senegal* or Seychelles* or "Sierra Leone" or Somalia* or "South Africa" or "South Sudan" or Sudan* or Tanzania* or Togo* or Tunisia* or Uganda* or Zambia* or Zimbabwe*).tw,id. |  |
| 12 | or/10-11 |  |
| 13 | 3 and 9 and 12 |  |

## **DATABASE: CINAHL**

| S11 | S3 AND S7 AND S10 |
| --- | --- |
| S10 | S8 AND S9 |
| S9 | TI ( Africa* OR Algeria* OR Angola* OR Benin OR Botswana* OR "Burkina Faso" OR Burundi* OR "Cabo Verd*" OR "Cape Verd*" OR Comoros* OR Cameroon* OR "Central African" OR Chad* OR Congo OR "Cote d'Ivoire" OR "Ivory Coast" OR Djibouti* OR Egypt* OR Eritrea* OR "Equatorial Guinea" OR Eswatini* OR Ethiopia* OR Gabon* OR Gambia* OR Ghana* OR Guinea* OR Kenya* OR Liberia* OR Libya* OR Lesotho* OR Madagascar* OR Malawi* OR Mali* OR Mauritania* OR Mauritius* OR Morocco* OR Mozambique OR Namibia* OR Niger* OR Nigeria* OR Rwanda* OR "Sao Tome" OR Senegal* OR Seychelles* OR "Sierra Leone" OR Somalia* OR "South Africa" OR "South Sudan" OR Sudan* OR Tanzania* OR Togo* OR Tunisia* OR Uganda* OR Zambia* OR Zimbabwe* ) OR AB ( Africa* OR Algeria* OR Angola* OR Benin OR Botswana* OR "Burkina Faso" OR Burundi* OR "Cabo Verd*" OR "Cape Verd*" OR Comoros* OR Cameroon* OR "Central African" OR Chad* OR Congo OR "Cote d'Ivoire" OR "Ivory Coast" OR Djibouti* OR Egypt* OR Eritrea* OR "Equatorial Guinea" OR Eswatini* OR Ethiopia* OR Gabon* OR Gambia* OR Ghana* OR Guinea* OR Kenya* OR Liberia* OR Libya* OR Lesotho* OR Madagascar* OR Malawi* OR Mali* OR Mauritania* OR Mauritius* OR Morocco* OR Mozambique OR Namibia* OR Niger* OR Nigeria* OR Rwanda* OR "Sao Tome" OR Senegal* OR Seychelles* OR "Sierra Leone" OR Somalia* OR "South Africa" OR "South Sudan" OR Sudan* OR Tanzania* OR Togo* OR Tunisia* OR Uganda* OR Zambia* OR Zimbabwe* ) |
| S8 | (MH "Africa+") |
| S7 | S4 OR S5 OR S6 |
| S6 | TI ( ((Mental NEAR/3 (Health OR illness OR disorder*)) OR Schizo* OR anxiety OR depression OR depressive OR bipolar OR paranoia OR psychosis OR trauma OR PTSD OR mania OR ((affective OR Stress* OR personality OR somatization OR somatoform OR mood) NEAR/4 disorder)) ) OR AB ( ((Mental NEAR/3 (Health OR illness OR disorder*)) OR Schizo* OR anxiety OR depression OR depressive OR bipolar OR paranoia OR psychosis OR trauma OR PTSD OR mania OR ((affective OR Stress* OR personality OR somatization OR somatoform OR mood) NEAR/4 disorder)) ) |
| S5 | (MH "Mental Health") |
| S4 | (MH "Mental Disorders+") |
| S3 | S1 OR S2 |
| S2 | TI ( Stigma OR prejud* OR discriminat* OR alienat* OR stereotyp* ) OR AB ( Stigma OR prejud* OR discriminat* OR alienat* OR stereotyp* ) |
| S1 | (MH "Stereotyping") OR (MH "Stigma") OR (MH "Social Alienation") OR (MH "Prejudice") OR (MH "Homophobia") OR (MH "Social Isolation") |

**Scopus**

((Schizo* OR anxiety OR depression OR depressive OR bipolar OR paranoia OR psychosis OR "affective disorder" OR “Stress disorders” OR “trauma and stressor related disorders” OR “psychological trauma” OR “post-traumatic stress” OR “post-traumatic stress disorder” OR PTSD OR “traumatic disorders” OR “personality disorders” OR “somatization disorders”)) OR AB ((Schizo* OR anxiety OR depression OR depressive OR bipolar OR paranoia OR psychosis OR "affective disorder" OR “stress disorders” OR “trauma and stressor related disorders” OR “psychological trauma” OR “post-traumatic stress” OR “post-traumatic stress disorder” OR PTSD OR “traumatic disorders” OR “personality disorders” OR “somatization disorders”)) AND((Africa OR Algeria* OR Angola* OR Benin OR Botswana* OR "Burkina Faso " OR Burundi* OR " Cabo Verd* " OR " Cape Verd* " OR Comoros*OR Cameroon* OR " Central African” OR Chad* OR Congo OR " Cote d'Ivoire " OR " Ivory Coast " OR Djibouti* OR Egypt* OR Eritrea*OR “Equatorial Guinea” OR” Eswatini* OR Ethiopia* OR Gabon* OR Gambia* OR Ghana* OR Guinea* OR “Guinea Bissau” OR Kenya* OR Liberia* OR Libya* OR Lesotho* OR Madagascar* OR Malawi* OR Mali* OR Mauritania* OR Mauritius* OR Morocco* OR Mozambique OR Namibia* OR Niger* OR Nigeria* OR Rwanda* OR “Sao Tome” OR Senegal* OR Seychelles* OR " Sierra Leone " OR Somalia* OR “South Africa” OR “South Sudan” OR Sudan* OR Tanzania* OR Togo* OR Tunisia* OR Uganda* OR Zambia* OR Zimbabwe*)).

**Pub Med**

We use to search based on the topic: internalized stigma among people with mental illness in Africa.
